# Method to obtain a plasma rich in platelet- and plasma-growth factors based on water evaporation

**DOI:** 10.1371/journal.pone.0297001

**Published:** 2024-02-21

**Authors:** Jon Mercader Ruiz, Maider Beitia, Diego Delgado, Pello Sánchez, Miren Begoña Sánchez, Jaime Oraa, Fernando Benito-Lopez, Lourdes Basabe-Desmonts, Mikel Sánchez

**Affiliations:** 1 Arthroscopic Surgery Unit, Hospital Vithas Vitoria, Vitoria-Gasteiz, Spain; 2 Microfluidics Cluster UPV/EHU, BIOMICs Microfluidics Group, Lascaray Research Center, University of the Basque Country UPV/EHU, Vitoria-Gasteiz, Spain; 3 Advanced Biological Therapy Unit, Hospital Vithas Vitoria, Vitoria-Gasteiz, Spain; 4 Microfluidics Cluster UPV/EHU, Analytical Microsystems & Materials for Lab-on-a-Chip (AMMa-LOAC) Group, Analytical Chemistry Department, University of the Basque Country UPV/EHU, Leioa, Spain; 5 Basque Foundation of Science, IKERBASQUE, Bilbao, Spain; Mie University Graduate School of Medicine, JAPAN

## Abstract

Platelet-Rich Plasma, also known as PRP, is an autologous biologic product used in medicine as a treatment for tissue repair. Nowadays, the majority of PRP obtention methods enrich only platelets, not considering extraplatelet biomolecules, which take part in several cell processes. In the present work, a novel PRP preparation method was developed to obtain a PRP rich in both platelet and plasma extraplatelet molecules. The method is based on the evaporation of the water of the plasma using a rotary evaporator. With this new methodology an increase in plasmatic growth factors and, as a consequence, a better dermal fibroblast cell viability was achieved, compared to a standard PRP formulation. This novel PRP product obtained with this new methodology showed promising results *in vitro* as an improved PRP treatment in future application.

## Introduction

Platelet-Rich Plasma (PRP) is a biological hemoderivative product obtained from blood in which platelets are present in a higher concentration than basal levels. PRP is applied in the treatment of different pathologies to promote tissue regeneration and reparation. These pathologies range from musculoskeletal disorders, oral and maxillofacial surgery and in dermatology, among others [1–4]. Due to its easy preparation, its low-cost processing and its autologous nature [5], PRP has gained great value as a treatment in regenerative medicine in the last few years [6].

Its restorative potential is assumed to be mainly based on platelets, which are anucleate blood elements, with a diameter of approximately 3 μm and derived from the hematopoietic line via the megakaryocyte, having a key role in hemostasis and thrombosis [7]. Platelet response to tissue damage occurs in several stages, starting with the adhesion of platelets to the extracellular matrix by the von Willebrand factor (VWF), expressed at the site of vessel damage, through its binding with the glycoprotein GPIbα, recruiting other platelets and activating them [8–10]. These activated platelets change their shape, modifying the conformation of the membrane protein GPIIb/IIIa, translocating functional proteins such as P-selectin, and releasing an arsenal of potential regenerative and mitogenic substances that are involved in wound healing and that play a key role in tissue regeneration. These substances immediately bind to the external surface of cell membranes in the graft, flap, or wound, via transmembrane receptors in mesenchymal stem cells, osteoblasts, fibroblasts, endothelial cells, and epidermal cells [11]. They mediate many of the cellular functions including cell migration, differentiation, cellular cycle, apoptosis and metabolism, as well as proliferation [12–14]. Therefore, platelets are expected to stimulate injured tissues and to regulate local inflammatory processes [11]. These released molecules include growth factors (GFs) and cytokines contained mostly in α-granules inside platelets [15]. The vast majority of factors that can be found within these granules are transforming growth factor (TGF-β1), platelet-derived growth factor (PDGF), fibroblast growth factor (FGF), epidermal growth factor (EGF) and vascular endothelium growth factor (VEGF). In addition to all these compounds derived from platelet activation, PRP also contains extraplatelet biomolecules in plasma with important biological activity. Among these are GFs such as the hepatocyte growth factor (HGF) or the insulin like growth factor (IGF-1), which are derived to a greater extent from the liver than from platelets [16, 17] and take part in the regulation of the chemotaxis, cellular differentiation and mitogenesis [18]. Moreover, these factors stimulate mesenchymal and epithelial cells to increase the synthesis of collagen and matrix to promote the formation of fibrous connective tissue and scar formation [14]. Besides molecules, structures such as microvesicles and exosomes also circulate within the plasma, being key in processes related to cell communication and signaling [19]. Therefore, despite the importance of platelets and their derivates, other extraplatelet components must be carefully considered in the therapeutic potential of PRP.

At present, the majority of the methods to obtain PRP focus on optimizing platelet concentration, with centrifugation being the most commonly used technique [20]. Thus, there are a large number of protocols by which different PRP products with a wide range of platelet, leukocytes and red blood cell concentrations can be obtained. This is based on different centrifugation protocols in which the time, speed and number of cycles are modified. Additionally, different method of activation can be used [20]. However, none of these methods address the concentration or modulation of the abovementioned extraplatelet plasma molecules, overlooking the possible biological activity they can bring to current PRPs.

Accordingly, we hypothesize that the enrichment of not only the platelet GFs but also the extraplatelet elements could lead to an improvement in the therapeutic capacity of PRP. Therefore, we report a new PRP production method based on water evaporation using rotary evaporator, achieving a novel PRP (nPRP) enriched in both platelets and plasma biomolecules, and whose composition and bioactivity were analyzed *in vitro*.

## Materials and methods

### Donors

Eight healthy donors were selected ranging in age from 30 to 72 years old, recruited between 8 September 2022 and 17 March 2023. Whole blood was withdrawn into tubes of 9 mL and 3.5 mL containing 3.8% (w/v) sodium citrate. 3.5 mL tubes were used to measure platelet concentration at baseline levels while the others were used to obtain the sPRP and the nPRP. Ethical approval was obtained from Ethics Committee of UPV/EHU (2019‐234, 13/05/2020) and written consent was obtained from patients.

### Standard Platelet-Rich Plasma (sPRP) preparation

The sPRP was obtained by centrifugation of 9 mL of blood at 580 x g for 8 min at room temperature to obtain the sPRP fraction, after collecting 2 mL of plasma fraction present over the red blood cell fraction (avoiding collecting white blood cells from the buffy coat). 10% CaCl_2_ (20 μL mL^-1^) was added to the plasma, to trigger platelet activation and clot formation.

### Novel Platelet-Rich Plasma (nPRP) preparation

**Fig 1:**
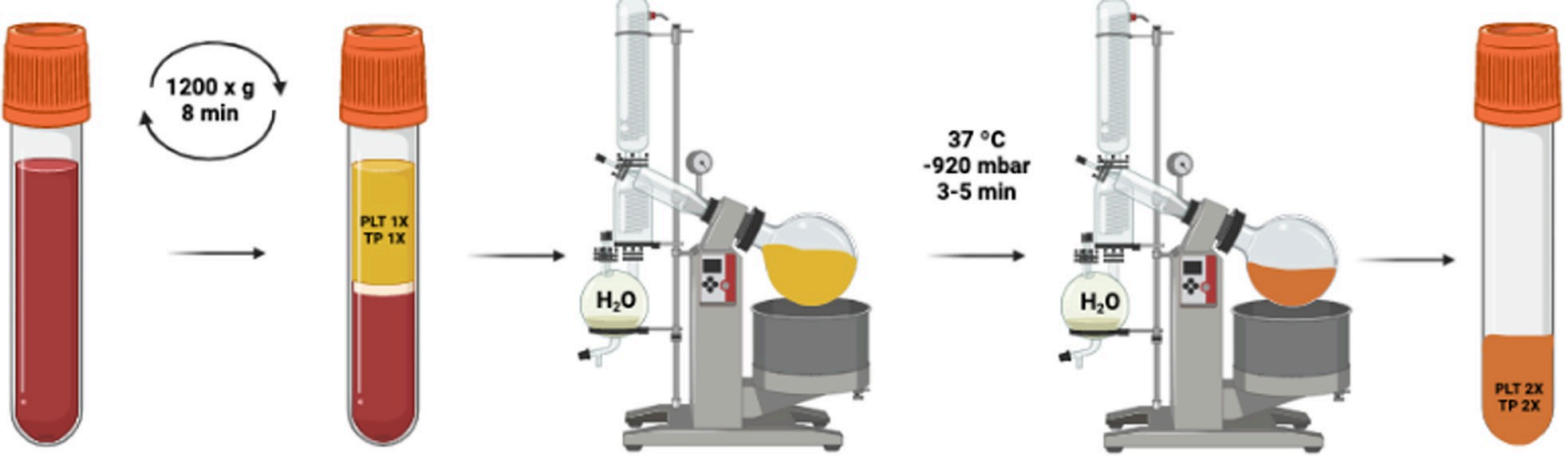
Schematic representation of the process to obtain nPRP using a rotary evaporator. PLT 1X represents similar levels of platelets comparing to basal levels, while TP 1X represents total proteins at basal levels. After the evaporation, both PLT and TP are concentrated, doubling its concentration.

The nPRP (Fig 1) was obtained by centrifugation of 9 mL of whole blood at 1200 x g for 8 min at room temperature, collecting a 4 mL plasma column from each tube, with no red or white blood cells. These centrifugation parameters were set to achieve a plasma column with both platelet (PLT) and total protein (TP) levels similar to those obtained in blood.

The next step was to remove the water from that plasma fraction in order to achieve the concentration of all its components. A rotary evaporator (Buchimodel R-100, Flawil, Switzerland) was used to evaporate half of the plasma water volume. The plasma column was transferred to a 100 mL round-bottom flask before assembling it to the rotary evaporator. The evaporation was performed for 3–5 min to evaporate half of the sample volume. A vacuum of—920 mbar and a temperature of 37°C was applied to the sample to decrease the boiling point and to improve evaporation, applying a similar temperature to that of the human body.

Finally, 10% CaCl_2_ (20 μL mL^-1^) was added to the plasma, to trigger platelet activation and clot formation, after restoring the pH at several values by adding HCl 0.8 M.

### Hematology and protein parameters

Platelet concentration and protein concentration were measured in whole blood, sPRP and nPRP samples from all the donors. Platelets were measured with a hematology analyzer (Sysmex XS-1000i; Kobe, Japan) whereas total protein levels were measured by a Cobas c 501 analyzer (Roche, Basel, Switzerland).

### Ion concentration analysis

Ion concentration measurements were carried out for sPRP and nPRP plasma samples by a Cobas c 501 analyzer (Roche, Basel, Switzerland). Ca^2+^, Na^+^, Cl^-^, Mg^2+^, P^3-^ and K^+^ were the selected ions to be quantified.

### pH and plasma coagulation time

After initial measurement of the pH of the nPRP obtained, the pH of the samples was adjusted with HCl to pH 8 and pH 7.4. Plasma clotting time was evaluated at the different pH values. pH measurements were performed by a Hanna pH-meter with a HI1331B electrode (Hanna Instruments, Italy).

### Platelet activation test

Measurement of P-selectin was used as a method to test the platelet activation. P-selectin, also called CD62P, is stored in α-granules of inactivated platelets. After platelet activation, the inner walls of these granules are exposed on the outside of the cells, presenting the CD62P protein [21]. For that purpose, CD41 and CD62P antibodies, which recognize platelet membrane constitutive glycoprotein GpIIb and translocated p-selectin respectively, were used.

For each donor, sample (10 μL), anti-CD41-FITC (5 μL) and anti-CD62P-PE (5 μL) (BD Biosciences, San Jose, CA, USA) were added in the test tubes. Afterward, 11 μL of the stimulating mixture (containing 5 μM of ADP (Sigma-Aldrich, Burlington, MA, USA)) in PBS were dispensed into the tubes corresponding to stimulating condition. The stimulating mixture was replaced by the same volume of PBS for the tubes used to measure the platelet activation at rest. Finally, samples were incubated for 15 min at RT in the dark and then were fixed with freshly prepared formaldehyde (400 μL, 1.25%) (PanReac AppliChem, Spain) in PBS (Gibco, Billings, MT, USA). A Gallios flow cytometer (Beckman-Coulter, High Wycombe, UK) was used to analyze events.

### Quantification of GFs

Both platelet and plasmatic GFs such as IGF-1, HGF and PDGF-AB, present in sPRP and nPRP were analyzed as follows. First, plasma samples were activated adding CaCl_2_ as mentioned above. Then, the concentration of those GFs was measured by commercially available enzyme-linked immunosorbent assay (ELISA) kits (R&D Systems, Minneapolis, MN, USA).

### Cell culture

Normal human dermal fibroblast (NHDF) (Lonza, Basel, Switzerland) were kept in the incubator at 37°C and 5% CO2 atmosphere. Cells were grown in fibroblast growth basal medium (FBM, Lonza, Basel, Switzerland) supplemented with insulin, human fibroblast growth factor, and gentamicin sulphate-amphotericin at 0.1% (v/v) each (Lonza, Basel, Switzerland), as recommended by the manufacturer.

### Cell viability assay

For the evaluation of the biological activity of both sPRP and nPRP, NHDF were incubated with FBM medium supplemented with 10% of either sPRP or nPRP platelet lysates, and cellular viability was registered at 96 h. Cellular viability was measured in triplicate for each donor by a Realtime‐Glo MT Cell Viability Assay (Promega, Fitchburg, MA, USA) that bases on the reducing potential of metabolically active cells that catalyze the conversion of a synthetic substrate into a luminescent product. Luminescence reading was performed by a TECAN Infinite 200 PRO plate reader (TECAN, Zurich, Switzerland). The level of luminescence can be considered proportional to the number of viable cells present in the assay [22]. Then, the obtained growth activity, with the different plasma formulations, were compared to see the proliferation rate of the nPRP compared with the sPRP.

### Statistical analysis

Distribution of the samples was assessed by Shapiro-Wilk’s normality test. The different variables were determined by the mean and the standard deviation for parametric data, and the median and 95% confidence interval (CI) for non-parametric data. Comparisons were performed by ANOVA and Student’s t-test for parametric data, and Kruskal-Wallis and Mann-Whitney U test for non-parametric data. Data were considered statistically significant when p<0.05. GraphPad Prism® software (San Diego, CA, USA) was used for the statistical analysis.

## Results

### Characterization of the nPRP

**Fig 2:**
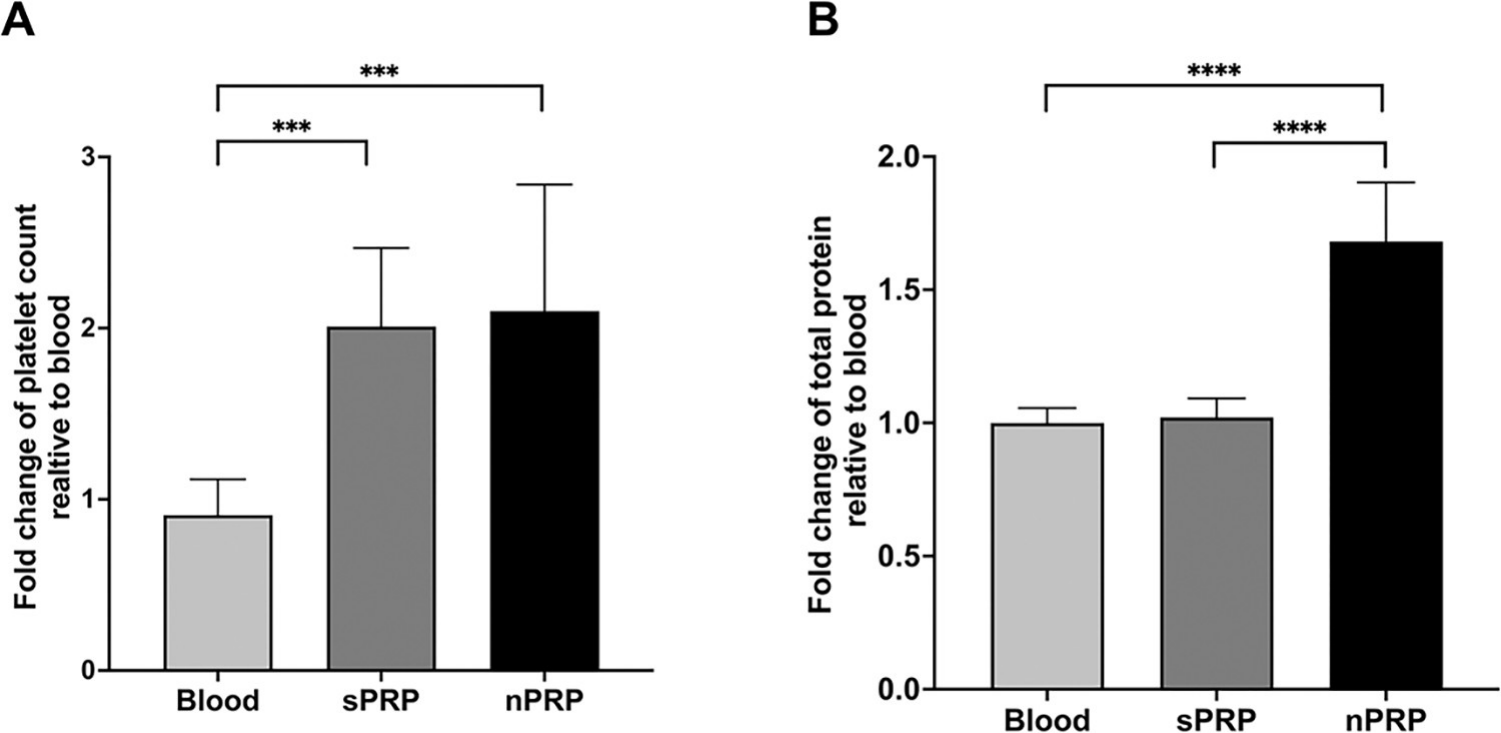
Platelet and total protein concentration levels in standard and novel PRP. Fold change values of (A) platelets and (B) proteins in blood and standard and novel PRP are shown. Error bars = standard deviation of eight donors. Statistically significant differences were calculated by Kruskall-Wallis and Dunn’s multiple comparison test post hoc for the platelet content and one-way ANOVA and Tukey’s multiple comparison test post hoc for total protein content (*** p<0.001; **** p<0.0001).

Both, sPRP and nPRP, showed a platelet concentration between 1.5 to 3 times higher than blood, reaching statistical significance (p = 0.0004 and p = 0.0007, respectively). However, no significant differences were found between them (Fig 2A). According to the classification and coding system made by Kon et al. [23], both plasmas have the classification code 13-00-11. This code is a sequence of 6 digits grouped in pairs indicating parameters of platelet composition, purity, and activation with the aim of unifying the way PRP is classified for comparison.

In terms of total protein concentration (Fig 2B), there was no statistical significance between blood and sPRP, whereas nPRP total protein concentration was almost doubled, comparing to whole blood and sPRP, being these differences statistically significant (p<0.0001 in both cases).

### Ion concentration capacity of sPRP and nPRP

Table 1 shows the concentration of the different salts present in blood, sPRP and nPRP samples. Regarding nPRP, due to water evaporation, all the ion levels increased doubling their concentration, reaching statistical significance when comparing them with blood and sPRP samples.

**Table 1:** Ions concentration present in blood, sPRP and nPRP. Calcium, sodium, chlorine, potassium, phosphorus and magnesium ion levels are represented. Values are expressed as mean ± standard deviation of eight donors. For the statistics ANOVA and Kruskal-Wallis test were done. Statistically significant differences are shown in bold.

Ion	Ion concentration			p value (a,b)	p value (a,c)	p value (b,c)
	Blood (a)	sPRP (b)	nPRP (c)			
**Ca^2+^ (mg dL^-1^)**	7.7 ± 0.2	8.1 ± 0.5	15 ± 2	>0.9999	**<0.0001**	**0.0021**
**Na^+^ (mmol L^-1^)**	161 ± 3	166 ± 7	307 ± 26	>0.8065	**<0.0001**	**<0.0001**
**Cl^-^ (mmol L^-1^)**	80 ± 4	84 ± 11	172 ± 21	>0.9999	**0.0006**	**0.0003**
**K^-^ (mmol L^-1^)**	3.4 ± 0.4	3.5 ± 0.3	6.3 ± 0.7	>0.7723	**<0.0001**	**<0.0001**
**P^3-^ (mg dL^-1^)**	3.0 ± 0.4	2.9 ± 0.4	5.3 ± 1	>0.9404	**<0.0001**	**<0.0001**
**Mg^2+^ (mg dL^-1^)**	2.1 ± 0.1	2.1 ± 0.1	3.9 ± 0.4	>0.9999	**<0.0009**	**<0.0001**

### Plasma pH adjustment and its impact in clotting time

**Fig 3:**
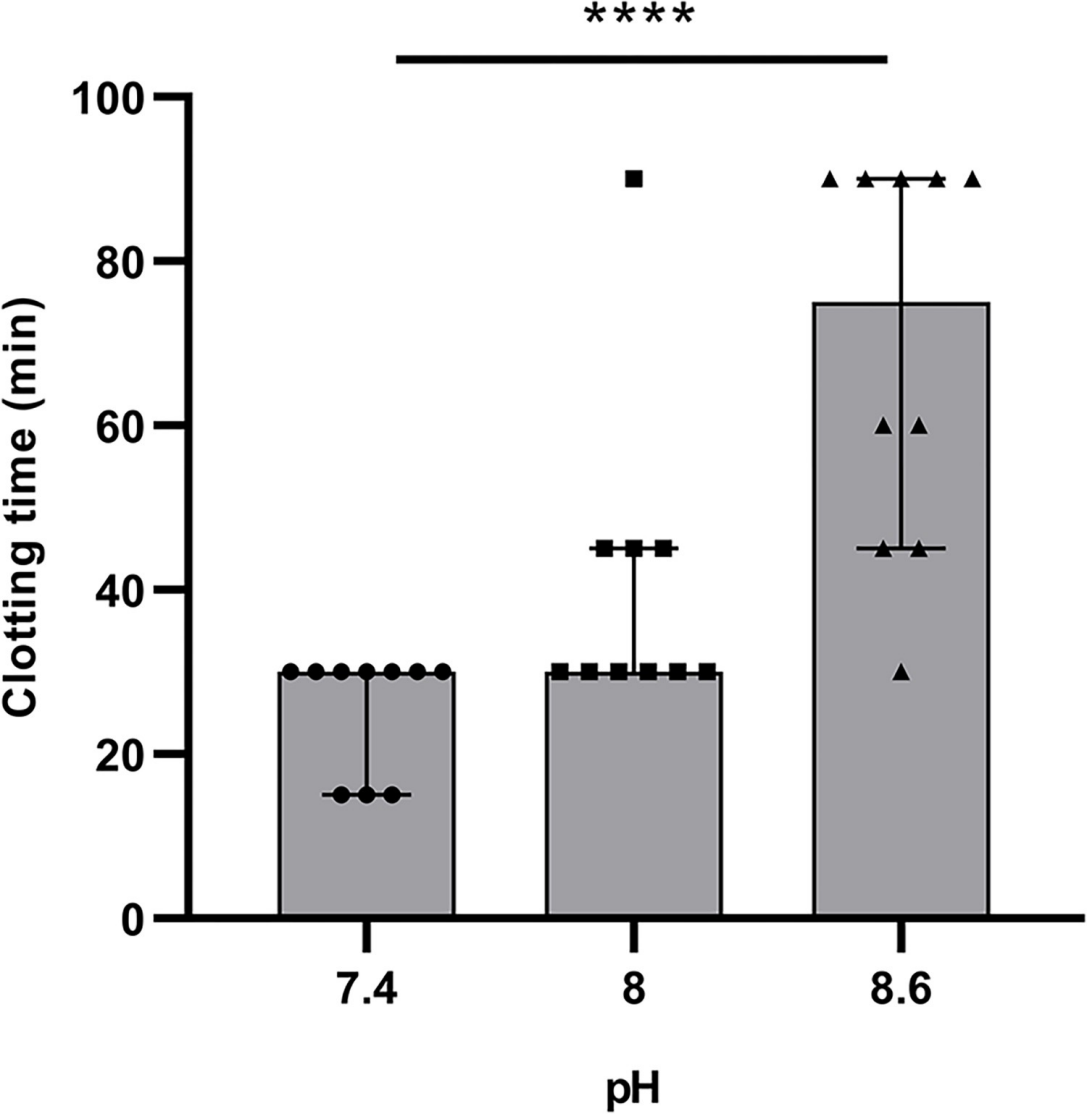
nPRP clotting time subject to sample pH. Different pH values are represented considering the pH of the nPRP. From pH 8.6, and after decreasing the sample to pH 8 and to physiological values with HCl solution. Values expressed as median ± 95% CI of eight donors. For the statistics, Kruskal-Wallis test was done with Dunn’s multiple comparison post hoc (****p < 0.0001).

In all cases, the sPRP showed a pH of 7.4 and the fibrin clot was formed during the first half hour after the addition of calcium chloride. However, the nPRP showed a higher pH and longer clotting times. The pH of the nPRP, 8.6, was reduced with an HCl solution to pH 8 and pH 7.4 in order to see if the clotting time of the plasma is reduced as the sample is acidified to baseline values. In fact, at pH 8.6 the coagulation used to take place after 60 min, generating a clot with a flimsy form. According to Fig 3, as the pH was reduced to physiological values (pH 7.4), the clotting time of the plasma decreased, being a significant difference in clotting time when it was reduced from pH 8.6 to physiological levels (p<0.0001).

### sPRP and nPRP impact in platelet activation

**Fig 4:**
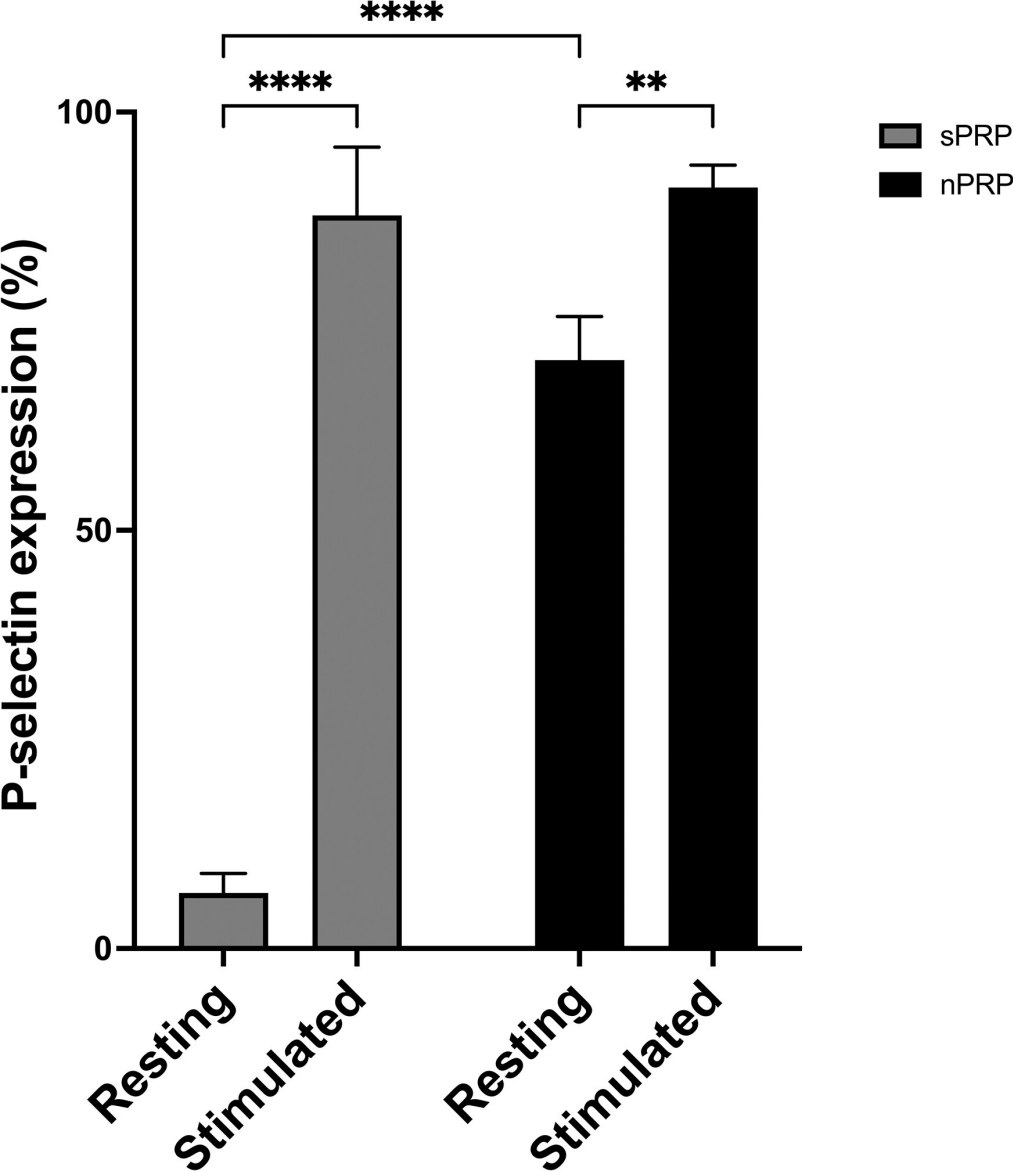
Platelet activation in standard and novel PRP. The graph represents the percentage of positive cells that are indicative of activated platelets (resting condition) and platelets activated by adding ADP as a platelet activator (stimulate condition). Values expressed as mean ± standard deviation of eight donors. Statistical significance was calculated by two-way ANOVA (**p < 0.01; ****p < 0.0001).

In addition, the level of platelet activation that was produced during the obtaining of each of the plasmas was analyzed. The analysis was based on the measurement of the expression of CD62 or p-selectin (marker of activated platelets). The results showed that when platelets were stimulated with ADP, activation reached 87% and 91% in sPRP and nPRP, respectively. On the other hand, the resting samples showed activation levels of 7% and 70%, respectively. Statistically significant differences were observed between the resting and stimulating condition in both PRPs (p<0.0001 in sPRP; p<0.01 in nPRP) as well as between the sPRP and nPRP at rest (p<0.0001) (Fig 4).

### Growth factor profile in sPRP and nPRP

**Fig 5:**
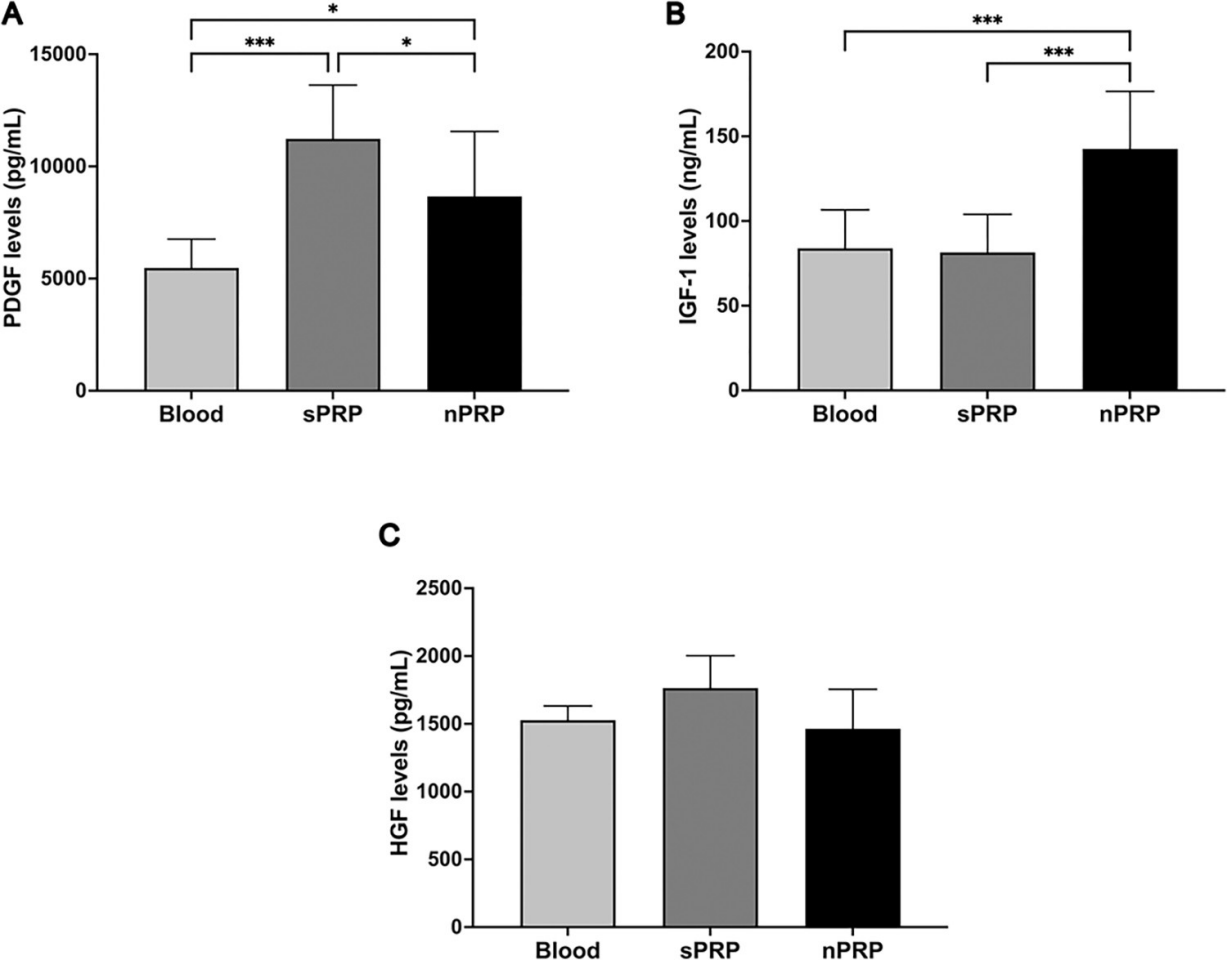
Growth factor levels presented in blood, sPRP and nPRP. Platelet (A) PDGF and plasmatic (B) HGF and (C) IGF-1 growth factor concentration levels are expressed as mean ± standard deviation of eight donors. Statistical significance was calculated by one-way ANOVA and Tukey’s multiple comparison test post hoc (*p < 0.05 and ***p < 0.001).

In order to analyze the levels of platelet and plasmatic GFs in the nPRP, enzyme‐linked immunosorbent assays (ELISAs) were performed in the platelet lysates of eight donors. A platelet growth factor PDGF and two plasmatic GFs (HGF and IGF-1) were analyzed (Fig 5). Both sPRP and nPRP showed a significant increase in PDGF levels compared to blood (p = 0.0003 and p = 0.0117, respectively). However, this enrichment was greater in sPRP than in nPRP (p = 0.0437). In the case of IGF-1, only nPRP showed a significant increase in IGF-1 levels compared to blood and sPRP (p = 0.0004 and p = 0.0005, respectively). Finally, the HGF factor, present both inside and outside platelets, was not enriched in either sPRP or nPRP compared to blood.

### Cell proliferation capacity of sPRP and nPRP

**Fig 6:**
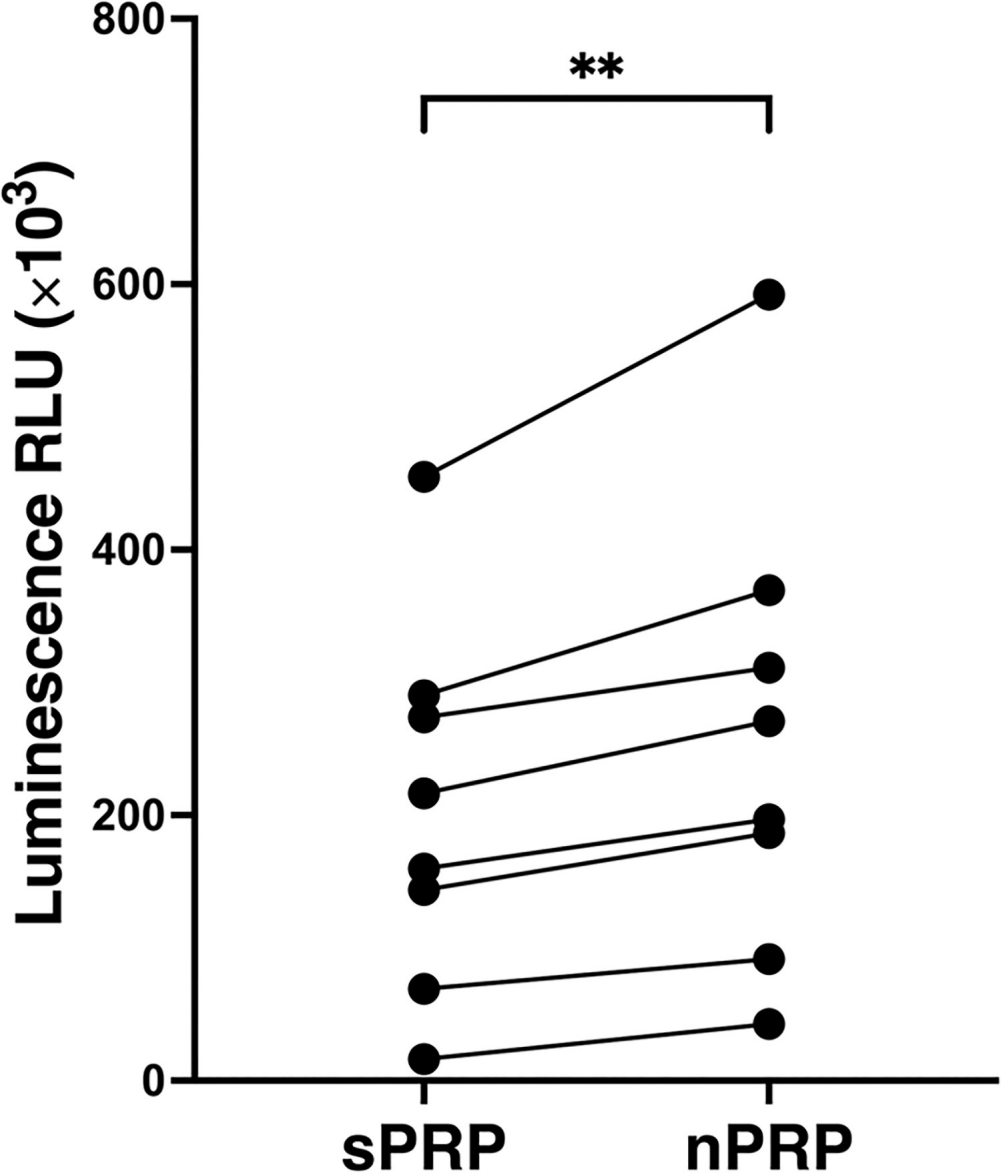
NHDF cell proliferation analysis. The viability levels of the cells incubated with sPRP and nPRP are expressed as relative lights units (RLU) and each point represents a different donor (n = 8). Statistical analysis was calculated by t-test (** p < 0.01).

The bioactivity of the nPRP was performed at 96h. NHDF cultures were exposed to sPRP and nPRP of eight different donors. In all the cases the bioactivity was measured comparing the cell growth of both plasma formulations, sPRP and nPRP. NHDF cultures showed statistically significant higher proliferation rate when treated with nPRP than when treated with sPRP (p = 0.0046) (Fig 6). The differences between sPRP and the nPRP were statistically significant after 96 h.

## Discussion

Over the past few years, PRP-based therapies have spread rapidly in various fields of medicine. Its easy production and high biosafety make it a very accessible therapy with promising results [2, 24]. However, during all these years of PRP development, there have not been any major breakthroughs in this technology [25]. The obtaining methods are very similar to each other and are based on centrifugation of blood to concentrate platelets. One of the main drawback of these systems is that they are not able to concentrate extraplatelet molecules and, thus, do not exploit its therapeutic potential [26, 27]. The main characteristic of the presented nPRP, and which differentiates it from other systems, is that by evaporating and removing the water from the plasma, not only the platelets are concentrated but also all the biomolecules that circulate outside them. In this manner, a balance in the concentration of both plasmatic and platelet GFs is achieved.

Starting from a plasma fraction with the same basal levels of both proteins and platelets than in whole blood, it was possible to double their concentration by evaporating half of the volume of water of the sample at physiological temperature (37°C). At these mild experimental conditions, proteins are protected from denaturation, as temperatures above 43°C were proven to denature proteins present in plasma [28].

As a result of this concentration increase, an elevation ion levels were also observed. The concentration of the ions presents in the plasma are crucial to keep the pH of the sample stable. Alteration of the basal concentration of these ions can produce powerful electrolytic forces due to imbalanced Cl^-^/Na^+^ concentrations, affecting the pH of the plasma [29], potentially implying a disadvantage. In fact, alteration of the basal concentration of ions can produce an imbalance between strong cations like sodium, calcium, potassium and magnesium and strong anions like chlorine. The strong ion difference (SID) is the difference between the sum of all strong cations and all strong anions. SID has a powerful electrochemical effect on water dissociation, and hence on H^+^ concentration. As SID becomes more positive, H^+^, a ‘weak’ cation, decreases (and pH increases) in order to maintain electrical neutrality [29]. In fact, the pH of the nPRP raised to pH 8.6 ± 0.3 compared to the physiological sPRP at pH 7.4 [30]. That change in the pH could affect protein function, including those proteins that take part in the activation of the coagulation cascade. Also fibrin network could suffer significant changes due to the variation in the ionic strength and pH, and to the high concentration of calcium ions, affecting the transformation of fibrinogen into fibrin [31–33]. In addition, an increase in pH above basal blood levels can induce changes in platelet function and directly affect clotting mechanisms [34]. All these factors could lead to a longer clotting times, or even prevent the plasma from clotting. Therefore, it was necessary to readjust the pH to physiological levels with HCl in our method before adding CaCl_2_ to the sample to promote its coagulation, as calcium is an important factor in clot formation that acts as a cofactor of thrombin [35], being essential for the activation of the coagulation cascade. Thus, it was found that restoring pH to physiological levels resulted in normal coagulation, solving the drawback.

On the other hand, the novel methodology for PRP obtaining was able to enrich not only the platelet-derived GFs, but also the extraplatelet ones, in contrast to the sPRP. However, although platelets were enriched twice in the nPRP, this did not result in a doble platelet-derived factors concentration. This could be due to the fact that during the evaporation process, almost 70% of the platelets are activated, potentially as a result of the platelet-glass contact or even the vacuum applied during the PRP collection [36]. This premature activation could lead to a prompt release of certain GFs that may get stuck to the glass of the flask. In addition, it has been shown that premature platelet activation could affect the functionality of platelets and, in consequence, to the GF release content [37]. Regarding the plasmatic factors (IGF-1 and HGF), it was found that IGF-1 levels were doubled in nPRP, thus achieving an increase in the concentration of this plasmatic GF, which has been reported to stimulate collagen production, facilitate tendon healing or promote cell proliferation [38, 39]. Moreover, this could be beneficial in elderly patients as this GF decreases with age [40]. However, the results showed that the plasmatic factor HGF remained at baseline levels in the nPRP. This could be due to the adsorption of the proteins into the glass during evaporation at the rotary evaporator [41, 42]. During this process, the Vroman Effect would be generated, whereby the larger proteins displace the smaller ones from the surface of the material, resulting in a much more stable bond. Indeed, out of the growth factors analyzed, HGF is by far the one with the highest molecular weight (HGF = 89 KDa, PDGF = 26 KDa and IGF-1 = 7 KDa), which could explain why the absorption of this protein would be stronger than the rest [43]. However, future experiments would be needed to elucidate the lack of enrichment of certain GFs by using surface modified flask as the polyethylene glycol (PEG) coated flasks [44]. PEG is a potential agent against protein adsorption, which might prevent the adhesion of biomolecules to it and, thus, concentrate HGF.

Eventually, the effect of sPRP and nPRP on the proliferation of NHDF culture was compared. The results showed a higher proliferation capacity on those cells exposed to nPRP, despite an excess of ions. This proliferation response could be partly due to the fact that more IGF-1 was concentrated in the nPRP, which has a great mitogenic power, favoring cell growth and proliferation [18, 38, 39].

Among all the results obtained, despite the initial disadvantages presented due to the pH mismatch after water evaporation, the novel composition of this plasma has shown to possess greater bioactivity on cells than sPRP, making it a potential candidate for future applications in regenerative medicine. As a limitation, due to the great variability among patients, it would be necessary to increase the number of samples to obtain more statistical relevant results. However, clinical studies and other cellular processes such as cell migration or inflammation, among others, will be needed when facing a real applicability of the obtained plasma, in order to verify potential clinically associated improvements.

## Conclusions

The present study indicates that the method of evaporating water from a plasma sample using a rotary evaporator is suitable for the obtaining of a novel PRP with a higher concentration of both platelets and biomolecules. In contrast to the conventional PRPs, this method concentrates not only the GFs that are inside platelets but also the growth factor IGF-1, which is key in anti-inflammatory processes and has a high mitogenic potential. In addition, the excess of ions in the medium does not negatively affect the clotting process of the nPRP since, by manual adjustment of the pH, the coagulation process is carried out normally. Finally, the nPRP induces a higher proliferative capacity of NHDF cells compared to sPRP.
